# How Well Does the Family Longevity Selection Score Work: A Validation Test Using the Utah Population Database

**DOI:** 10.3389/fpubh.2018.00277

**Published:** 2018-10-01

**Authors:** Liubov S. Arbeeva, Heidi A. Hanson, Konstantin G. Arbeev, Alexander M. Kulminski, Eric Stallard, Svetlana V. Ukraintseva, Deqing Wu, Robert M. Boudreau, Michael A. Province, Ken R. Smith, Anatoliy I. Yashin

**Affiliations:** ^1^Thurston Arthritis Research Center, University of North Carolina, Chapel Hill, NC, United States; ^2^Department of Surgery, University of Utah, Salt Lake City, UT, United States; ^3^Population Sciences, Huntsman Cancer Institute, University of Utah, Salt Lake City, UT, United States; ^4^Biodemography of Aging Research Unit, Social Science Research Institute, Duke University, Durham, NC, United States; ^5^Department of Epidemiology, University of Pittsburgh, Pittsburgh, PA, United States; ^6^Division of Statistical Genomics, Department of Genetics, Washington University School of Medicine, St. Louis, MO, United States; ^7^Department of Family and Consumer Sciences, University of Utah, Salt Lake City, UT, United States

**Keywords:** exceptional survival, familial longevity, Long Life Family Study, Utah population database, family longevity selection score

## Abstract

The Family Longevity Selection Score (FLoSS) was used to select families for the Long Life Family Study (LLFS) but has never been validated in other populations. The goal of this paper is to validate how well the FLoSS-based selection procedure works in an independent dataset. In this paper, we computed FLoSS using the lifespan data of 234,155 individuals from a large comprehensive genealogically-based resource, the Utah Population Database (UPDB), born between 1779 and 1910 with mortality follow-up through 2012–2013. Computations of FLoSS in a specific year (1980) confirmed the survival advantage of the “exceptional” sibships (defined by LLFS FLoSS threshold, FLoSS ≥ 7). We found that the subsample of the UPDB participants born after 1900 who were from the “exceptional” sibships had survival curves similar to that of the US participants from the LLFS probands' generation. Comparisons between the offspring of parents with “exceptional” and “ordinary” survival showed the survival advantage of the “exceptional” offspring. Investigators seeking to explain the extent genetics and environment contribute to exceptional survival will benefit from the use of exceptionally long-lived individuals and their relatives. Appropriate ranking of families by survival exceptionality and their availability for the purposes of providing genetic and phenotypic data is critical for selecting participants into such studies. This study validated the FLoSS as selection criteria in family longevity studies using UPDB.

## Introduction

Exceptional survival is a combined outcome of many factors such as genetics, life style, and environmental exposures. Studying exceptionally long-lived individuals and their relatives in family longevity studies can help explain the extent that genetics and environment contribute to this trait. Selecting participants into such studies can be challenging because exceptional survivors represent a small fraction of a general population and, in addition, the studies often need to enroll living siblings (who can provide essential epidemiological, demographic, and biospecimen data) who should for practical reasons reside within the study area (also of note, there must be any siblings before there can be long lived siblings—hence the recruitment for exceptional longevity among siblings is a function of cohort fertility). In addition, the definition of “exceptionality” of familial longevity is important for constructing specific procedures for identifying the truly “exceptional families” enriched for longevity while ensuring a sample size with adequate power.

Sebastiani et al. (1) developed the Family Longevity Selection Score (FLoSS). This score was used to select the families for the Long Life Family Study (LLFS). It takes into account both exceptionality of family members' survival and the presence of very old living family members. The necessary information can be collected from living family members and used to compute the score. The selection procedure assumes that FLoSS generated from scores reflecting exceptional survival of individual family members will identify “exceptional” families. However, there is no “direct” way to test whether the resulting LLFS sample has better survival in relation to a general population.

In LLFS, all selected individuals were alive at the time of enrollment and a subsample of those survived until the end of the follow up period. This means that lifespan data for these surviving individuals are incomplete (right censored and left truncated). Therefore, “direct” testing for exceptional longevity of selected individuals would require additional time in order to see the cohorts become extinct. To test whether the procedure used for selecting LLFS participants identifies individuals with exceptional longevity, one can apply the same computational procedure using FLoSS to individuals in birth cohorts who have complete information about their lifespans. This allows one to determine whether survival functions for “exceptional” (“selected”) individuals are superior to those in the general population comprising individuals from the same birth cohorts but who were not selected by this procedure.

The goal of this paper is to validate how well the FLoSS-based selection procedure performs in an independent dataset. Here, we present results where we compute FLoSS using the lifespan data for a sample from the Utah Population Database (UPDB). This database is widely used for population-based analyses of familial risk and genetic susceptibilities that allow researchers to estimate the heritability of longevity and other phenotypes and identify predisposition genes responsible for the extreme life span (2, 3). In our application, these data provide the unique opportunity to test the hypothesis that the score used for LLFS recruitment identifies families with exceptional longevity by computing FLoSS based on data on lifespans of UPDB family members and comparing the survival curves of the “exceptional” and “ordinary” longevity UPDB subjects (as defined by the FLoSS thresholds similar to LLFS).

## Materials and methods

### Characteristics of UPDB subsample used to calculate FLoSS

This study utilizes data drawn from the Utah Population Database (UPDB). The UPDB is one of the world's richest sources of linked population-based information for demographic, genetic, and epidemiological studies. UPDB has supported numerous biomedical investigations in large part because of its size, inclusion of multi-generational pedigrees, and linkages to numerous data sources. The UPDB now contains data on over 11 million individuals from the late eighteenth century to the present and is representative of Utah's population. The holdings of the data grow due to longstanding efforts to update records as they become available including statewide birth and death certificates, hospitalizations, ambulatory surgeries, and driver licenses. UPDB creates and maintains links between the database and the medical records held by the two largest healthcare providers in Utah as well as Medicare claims. The multigenerational pedigrees representing Utah's founders and their descendants were constructed based on data provided by the Genealogical Society of Utah (GSU). Pedigrees spanning the past 80 years have been expanded extensively based on vital records and, together with the GSU data, form the basis of the deep genealogical structure of the UPDB (2, 4, 5). This study has been approved by the University of Utah's Resource for Genetic and Epidemiologic Research and its Institutional Review Board.

The subset of the UPDB available for this study (referred to as the “UPDB sample” throughout the text) contains information on individuals from UPDB families that fulfill the following requirements: (1) an individual and all siblings are born in 1910 or earlier; (2) an individual and all siblings have information on a year of birth and a year of death or a last living year; (3) an individual's mother and father have information on birth and death years or a last living year. The resulting sample contains 400,822 individuals born between 1779 and 1910 with follow-up data through 2012–2013. Among them, 326,023 individuals have known information about lifespan (computed as year of death minus year of birth), with the remainder having a last known living year.

### Computation of FLoSS in UPDB sample

The methodology for calculating FLoSS in LLFS is described in detail in Sebastiani et al. (1) (see also Supplementary Material). We computed FLoSS in the UPDB sample by adopting this methodology. In Sebastiani et al. (1), exceptional longevity of individuals was calculated based on birth year- and gender-specific cohort survival probabilities from the US Social Security Administration (SSA) cohort life tables. Since such life tables are not available for cohorts earlier than 1900, in this analysis we constructed gender-specific cohort life tables (for 10-year birth cohorts) using available lifespan data from the UPDB sample. All individuals born before 1850 were grouped into one cohort due to its modest sample size. We excluded adopted siblings and family members who died before age 40, similar to the restriction used with the LLFS procedure. The resulting sample (“UPDB-All”) contained 234,155 individuals. These conditional (at age 40) cohort life tables from the UPDB sample were used for calculation of FLoSS as described in Sebastiani et al. (1). We constructed different subsamples from the UPDB-All sample relevant for our analyses as described below (see also Supplementary Table 1).

We used the same threshold as in LLFS (FLoSS ≥ 7) to select exceptional sibships in our study (the “UPDB-FLoSS” sample). In LLFS, this threshold was chosen because it was determined that such families are rare but are still detectable with sufficient frequency (1). In our applications, this threshold corresponds approximately to the highest 2.2% of the FLoSS distribution among the UPDB participants born after 1900 (referred to as the “UPDB-1900” sample; the subset of this sample with FLoSS ≥ 7 is denoted as the “UPDB-1900-FLoSS” sample). This particular year was selected as one of the earliest birth cohorts in LLFS data and is also the earliest year for which the US SSA cohort lifetables are available. We also selected sibships with FLoSS ≤ −4.5 which is approximately the lowest 2.2% of the FLoSS distribution among the UPDB participants born after 1900 (the corresponding sample is referred to as “UPDB-1900-AntiFLoSS”).

We note that this study cannot fully reproduce the LLFS design and selection procedure (see Discussion) because, for example, the latest birth cohort in the UPDB sample is 1910, therefore, there are no offspring of individuals born after 1900 (which is the range of birth cohorts similar to LLFS).

### Computations for cross-sectional time point

The wide range of birth cohorts available in the UPDB sample allows selection of various cross-sectional time points (referred to as “time points” or “years of study” in the following text) for selection of “exceptional” families at such time points and following their subsequent survival. Selecting a time point closer to the LLFS baseline visit (which started in 2006), i.e., using data on later birth cohorts, would provide a better comparison with the LLFS probands' generation (the oldest generation in LLFS consisting of individuals from “exceptional” families identified by FLoSS and their spouses). However, this choice results in a smaller sample of individuals surviving until that time point (due to a range of birth cohorts limited by 1910 in the UPDB sample). Therefore, we used earlier time points to allow the inclusion of more individuals in the sample of survivors (see description in Results).

For a specific year of study, we recalculated age and vital status at this time point. That is, if year of death (or last known living year) was after the year of study, then we assigned the individual's vital status as “alive” and computed his/her age at this time point. We then selected all families with at least one living individual aged 80 or above in the study year to construct two subgroups, “exceptional” and “ordinary,” based on the FLoSS. The “exceptional” group contained individuals who were alive and aged 80 or above in the study year and belonged to families with FLoSS ≥ 7. All other individuals who were alive and aged 80 or above in the study year but were not selected in the “exceptional” group comprised the “ordinary” group (which would not be selected into LLFS).

We computed Kaplan-Meier estimates of the survival functions for the “exceptional” and “ordinary” groups using follow-up data on mortality for these individuals. Age at the study year was used as the left truncation variable. The log-rank test was used to evaluate the differences between survival curves.

### Computations with offspring of parents with “exceptional” and “ordinary” survival

The UPDB sample is multi-generational which allows analyses of survival patterns of offspring of parents with “exceptional” and “ordinary” survival as identified by FLoSS. We constructed two groups, the “exceptional offspring” group and the “ordinary offspring” group. We identified “exceptional offspring” as those who have either mother or father (or both) with FLoSS ≥ 7 and “ordinary offspring” as those with both parents from families with FLoSS < 7. We computed the Kaplan-Meier estimates of the survival functions for the “exceptional offspring” and “ordinary offspring” born after 1900 using follow-up data on mortality for these individuals.

All analyses were performed in SAS 9.4 and R 3.4.3. Graphical output was prepared in R and MATLAB R2017b.

The study performs secondary analyses of previously collected data. The study was approved by the Duke University Campus Institutional Review Board (protocol C0027) and Duke University Medical Center Institutional Review Board (protocol 10045).

## Results

### Survival exceptionality of UPDB-FLoSS sample

The FLoSS computation procedure was applied to the UPDB-All sample to identify families with exceptional FLoSS (FLoSS ≥ 7). There were 799 families with 5,684 individuals selected in the resulting UPDB-FLoSS sample. The average FLoSS in the UPDB-All sample of 57,192 sibships was M = −0.234 and standard deviation was S = 2.9. Supplementary Figure 1 shows distributions of standardized FLoSS (S-FLoSS), computed as S-FLoSS = (FLoSS-M)/S, in the UPDB-All and UPDB-FLoSS samples. Supplementary Figure 2 presents distributions of age at death for these samples.

**Figure 1:**
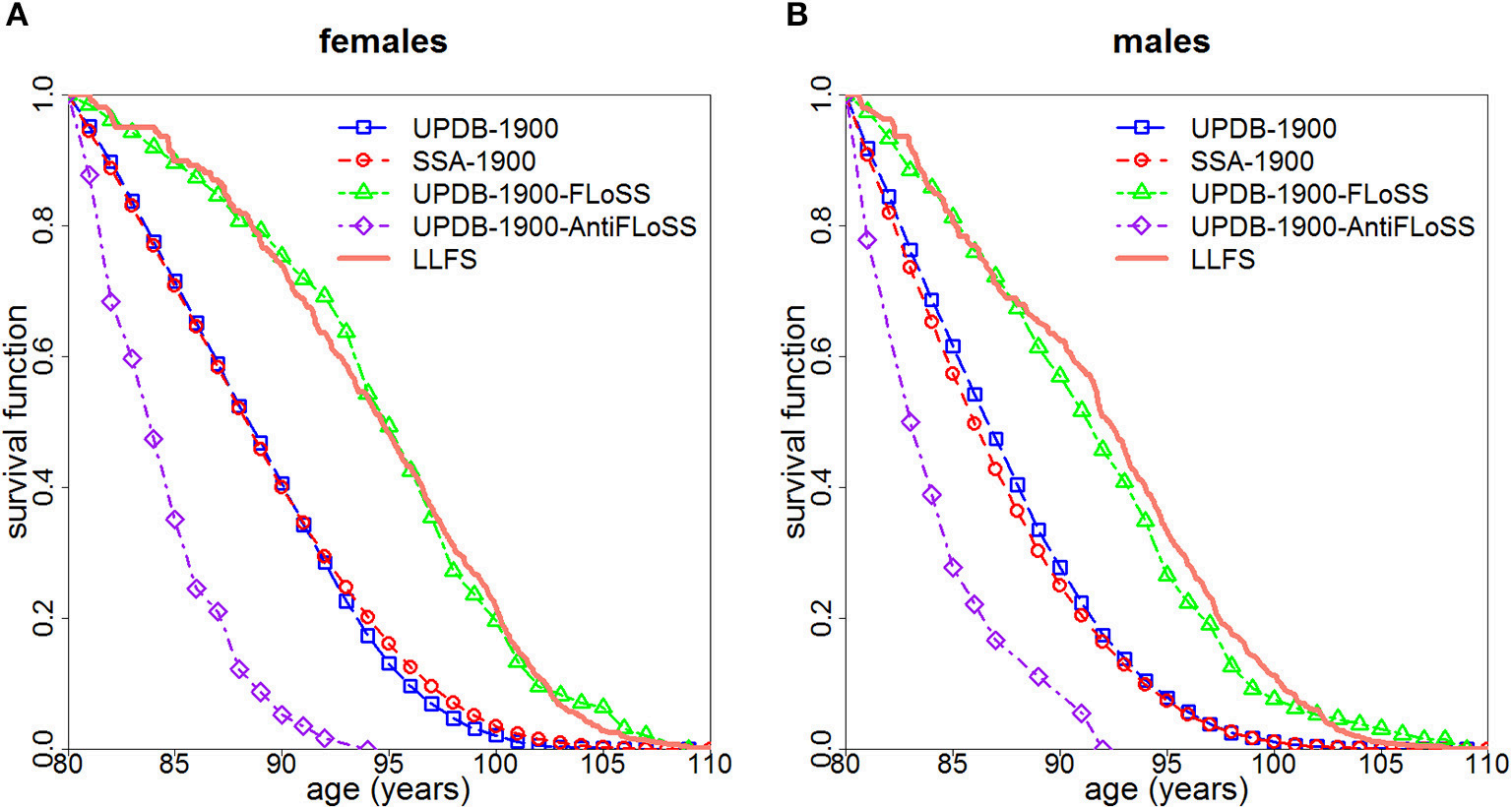
Survival curves (conditional at age 80) for females **(A)** and males **(B)**: (1) for all individuals from the UPDB sample born after 1900 (“UPDB-1900”); (2) based on the US SSA 1900 cohort life tables (“SSA-1900”); (3) for individuals from UPDB-1900 with FLoSS ≥ 7 (“UPDB-1900-FLoSS”); (4) for individuals from UPDB-1900 with FLoSS ≤ −4.5 (“UPDB-1900-AntiFLoSS”); (5) for the US participants from the probands' generation in LLFS (“LLFS”).

Figure 1 displays survival curves for the UPDB-1900, the UPDB-1900-FLoSS and the UPDB-1900-AntiFLoSS samples, along with survival curves based on the US SSA 1900 cohort life tables and the LLFS survival curves representing the Kaplan-Meier curves (conditional at age 80) computed for subsets of US participants of respective sex from the LLFS probands' generation based on the study sample assessed on March 13, 2017. Figure 1 shows that the UPDB-1900-FLoSS participants have survival that is very similar to the LLFS probands whereas the entire UPDB-1900 sample has a conditional (at age 80) survival curve that is also very similar to that of the general US population. The UPDB-1900-AntiFLoSS sample has substantially worse survival than the entire UPDB-1900 sample as expected.

### Calculations at a single time point

We constructed “exceptional” and “ordinary” groups as described in the Material and methods section for different time points from 1900 to 2000. Because of the specifics of the UPDB sample analyzed here (e.g., the maximal year of birth is 1910), the number of living individuals aged 80 years and above reached the maximum from the late 1970s to the early 1980s, decades that also provide the maximum number of individuals in the “exceptional” group. We selected 1980 as the representative “study year” for this analysis. At this point, 15,144 individuals were alive and 80 years or older and among those, 1,193 were classified as the “exceptional” group and the remaining 13,951 formed the “ordinary” group (denoted UPDB-y1980-E and UPDB-y1980-O, respectively).

**Figure 2:**
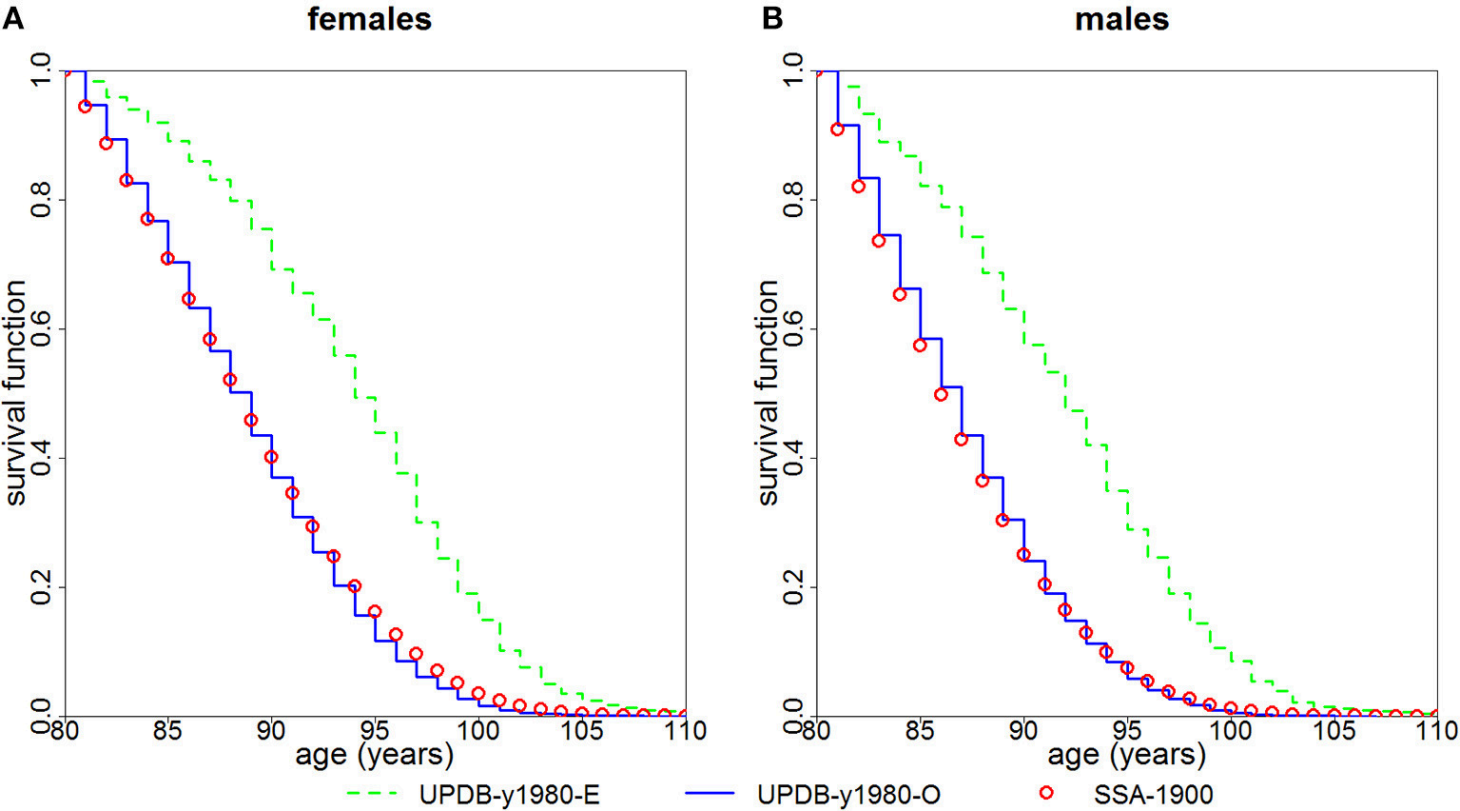
Kaplan-Meier estimates of survival functions for members of “exceptional” (“UPDB-y1980-E”) and “ordinary” (“UPDB-y1980-O”) groups for the study year 1980: **(A)** females and **(B)** males. The conditional survival curves (at age 80) computed from the US SSA 1900 cohort life tables (“SSA-1900”) are shown for comparison.

We followed both groups from year 1980 using the actual years of death/censoring. Figure 2 presents the Kaplan-Meier estimates of survival functions for the female and male members of the “exceptional” and “ordinary” groups. The figure shows that members of the “exceptional” group have better survival than the members of the “ordinary” group (*P* < 0.0001 for both females and males). It also shows that the survival of the “ordinary” group is quite similar to the US SSA 1900 cohort.

### Computations with offspring of parents with “exceptional” and “ordinary” survival

We selected the “exceptional offspring” and the “ordinary offspring” groups as described in the Material and methods section for offspring born after 1900 (denoted “UPDB-1900-EO” and “UPDB-1900-OO”, respectively). Altogether, 993 individuals (502 females, 491 males) were selected in the “exceptional offspring” group and 16,541 individuals (7,964 females, 8,577 males) were classified as the “ordinary offspring.” Among those, 587 (59.1%) “exceptional offspring” (347 females, 69.1%; 240 males, 48.9%) and 8,167 (49.4%) “ordinary offspring” (4,802 females, 60.3%; 3,365 males, 39.2%) survived until age 80.

**Figure 3:**
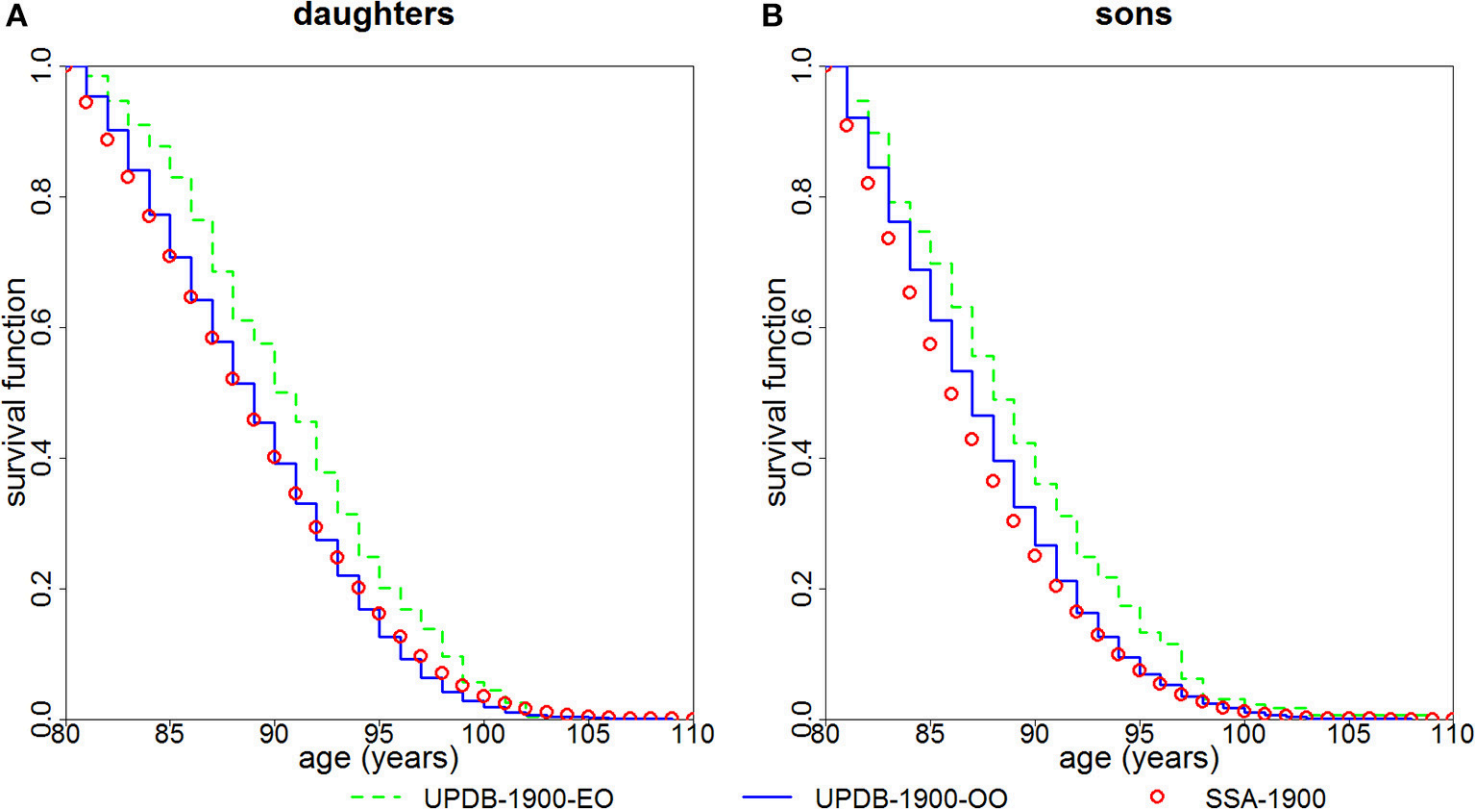
Kaplan-Meier estimates of survival functions (conditional at age 80) for offspring of parents with “exceptional” (“UPDB-1900-EO”) and “ordinary” (“UPDB-1900-OO”) survival: **(A)** females (“daughters”) and **(B)** males (“sons”). The sample contains offspring born after 1900. The conditional survival curves (at age 80) computed from the US SSA 1900 cohort life tables (“SSA-1900”) are shown for comparison.

Figure 3 shows survival functions (conditional on survival to age 80) for the “exceptional offspring” group and the “ordinary offspring” group born after 1900. The figure indicates that both daughters (*P* < 0.0001) and sons (*P* = 0.0003) of parents from the “exceptional” group have a survival advantage compared to offspring of parents from the “ordinary” group. Survival of the latter group is closer to that of the general population represented by the SSA 1900 cohort.

## Discussion

The unique advantages of the UPDB, including its comprehensive coverage of demographic data, extended sibship sizes and a wide range of birth cohorts allowed us to illustrate the potential benefit of using FLoSS as selection criteria in family longevity studies. Using FLoSS computed from information on lifespan in the UPDB sample, this study validated LLFS sample selection procedure based on FLoSS to identify families with exceptional longevity. Figure 1 illustrates that the subsample of the UPDB participants born after 1900 who were from sibships selected by the similar FLoSS threshold as in the LLFS (FLoSS ≥ 7) have survival curves (conditional on survival to age 80) remarkably comparable to that of the US participants from the LLFS probands' generation and both curves lie to the right of the “general” population curves (SSA and the full UPDB). We note also that the entire UPDB sample has a survival curve similar to that of the general US population despite the fact that Utah has more individuals affiliated with the Church of Jesus Christ of Latter-day Saints. These individuals generally do not use tobacco or consume alcohol so they are expected to have better survival. UPDB also includes fewer ethnic and racial groups who historically have poorer survival. However, conditional survival curves (at age 80) are presented here, therefore, any differences in survival manifested at younger ages are not represented in this figure. In addition, women in UPDB have more children which can attenuate the survival benefits of being members of the Church of Jesus Christ of Latter-day Saints. These and other factors can contribute to the observed patterns of cohort life expectancies in Utah and general populations [see discussion in (6)].

This study is limited because we cannot fully reproduce the LLFS enrollment procedure [see details, e.g., in Newman et al. (7)] due to data availability and limited sample sizes of the “exceptional” population. In LLFS, the proband was deemed qualified for the enrollment in the study if he/she had at least one living sibling and one offspring residing in the appropriate catchment area. These criteria were needed to provide additional relevant data for the analysis of phenotypic and genetic data. However, such criteria are not applicable to the UPDB sample. For example, the latest birth cohort is 1910 meaning that there are no offspring of “probands” born after 1900 (which is the range of birth cohorts similar to LLFS). Also, for FLoSS computations in the UPDB sample, we constructed cohort lifetables using the UPDB sample itself because SSA cohort lifetables (used in LLFS) are not available for cohorts earlier than 1900.

The advantage of this study is that in the UPDB sample we are able to locate the point in the distant past that yields the largest “exceptional” samples and follow them using the available data on mortality. Such computations in the UPDB sample further confirmed (Figure 2) that the members of the “exceptional” group (as defined by the FLoSS threshold) have better survival at old (80+) ages than the members of the “ordinary” group (i.e., those not selected in the “exceptional” group). We note that since age 80 is a more extreme survival threshold for men than for women, the narrower gap between the curves for men than for women in Figure 2 may be a function of men being closer to their maximum lifespan and hence having less room to improve upon over the SSA/UPDB ordinary survival.

The availability of multiple generations in the UPDB sample allowed us to compare survival of offspring of “exceptional” and “ordinary” groups. Figure 3 demonstrates that daughters and sons whose mothers and/or fathers are from the “exceptional” group have better survival compared to offspring whose both parents are from the “ordinary” group. However, the magnitude of the differences in survival is smaller than that between the “exceptional” and “ordinary” groups shown in Figure 2. This observation confirms the findings in other studies which also show that, in general, the correlation between the ages at death of siblings is stronger than the correlation between the ages at death of parents and children [see, e.g., (8, 9), among many others]. We also note that Utah males have more lifestyle differences than US males in general (relative to Utah females vs. US females) which can contribute to the result shown in Figure 3 where males deviate more from the SSA “standard” than females.

Family longevity studies provide opportunities to investigate relationships between various factors and longevity. The familial structure requires using specific approaches in analyses of such data. For example, frailty models can be used to analyze multigenerational studies on longevity to assess effects of unobserved environmental and genetic factors on longevity (9). Availability of genetic information in familial studies on longevity with follow-up information on mortality facilitates finding associations between heritable genetic markers and longevity. The studies show that using information on age at biospecimen collection in addition to follow-up data on mortality as well as incorporation of follow-up information on non-genotyped individuals can give substantial increase in power compared to analyses of follow-up data in genotyped individuals alone (10, 11). Such approaches can be extended to incorporate familial structure in the family longevity studies. Dynamics of various biomarkers is related to mortality and aging as the extensive literature documents (12) and availability of longitudinal measurements of relevant biomarkers in familial studies on longevity, coupled with appropriate statistical approaches, can substantially advance our knowledge on determinants of exceptional longevity.

This study demonstrates the value of familial and genealogical microdata captured for entire populations spanning many decades (13, 14). Increasingly these are available around the world (e.g., Sweden, Netherlands, and Quebec) and can be leveraged to identify families who are likely to harbor genetic variants associated with extreme ages at death. Linkages between these genealogies with comprehensive environmental and epidemiological data are also occurring and can help to sharpen the distinction between the influences of genetics and socio-demographic factors on exceptional human survival.

## Data availability statement

The Long Life Family Study (LLFS) data used in this study were provided by the LLFS Data Management and Coordinating Center. The LLFS data are also available in the database of Genotypes and Phenotypes (dbGaP) (https://www.ncbi.nlm.nih.gov/gap; dbGaP Study Accession: phs000397.v1.p1). The study also used a subset from the Utah Population Database (UPDB). Requests regarding access to the UPDB data should be directed to Ken R. Smith.

## Author contributions

LA analyzed data, wrote the paper. HH acquired and prepared data, wrote the paper. KA conceived and designed the study, analyzed data, wrote the paper. AK interpreted results and contributed to the final text. ES interpreted results and contributed to the final text. SU interpreted results and contributed to the final text. DW interpreted results and contributed to the final text. RB conceived and designed the study, interpreted results and contributed to the final text. MP conceived and designed the study, interpreted results and contributed to the final text. KS acquired data, wrote the paper. AY conceived and designed the study, interpreted results and contributed to the final text.

### Conflict of interest statement

The authors declare that the research was conducted in the absence of any commercial or financial relationships that could be construed as a potential conflict of interest.
