# Supplementary material for: How Well Does the Family Longevity Selection Score Work: A Validation Test Using the Utah Population Database
Source: Front Public Health. 2018 Oct 1;6:277. doi: 10.3389/fpubh.2018.00277 (PMC6174319; doi:10.3389/fpubh.2018.00277)
Supplement: Supplementary file 1 [file Data_Sheet_1.docx]

Supplementary Material

How Well Does the Family Longevity Selection Score Work: A Validation Test Using the Utah Population Database

Liubov S. Arbeeva, Heidi A. Hanson, Konstantin G. Arbeev*, Alexander M. Kulminski, Eric Stallard, Svetlana V. Ukraintseva, Deqing Wu, Robert M. Boudreau, Michael A. Province, Ken R. Smith, Anatoliy I. Yashin

*** Correspondence:** Konstantin G. Arbeev: [ka29@duke.edu](mailto:ka29@duke.edu)

# Supplementary Text: The Family Longevity Selection Score (FLoSS)

Technical details about the FLoSS are given in Sebastiani et al. (2009). Here we provide a brief overview of the main ideas of its computation.

As follows from its name, this score is defined for families as it serves the purpose of ranking families, not individuals. Nevertheless, the FLoSS is composed of the quantities characterizing individuals’ (i.e., family members’) “exceptionality of longevity” with an additional “bonus” for older living siblings, as described below. For a family member who died at some age, the individual’s “exceptionality of longevity” is characterized by the probability that a random person of the same gender from the same birth cohort survives to at least that age (transformed as a minus logarithm of the probability so that the respective quantity is positive; see also a probabilistic interpretation of this in (Sebastiani et al., 2009)). Such probabilities can be determined from respective country-, cohort-, and gender-specific life tables. In the US, such life tables are provided by the Social Security Administration (Bell and Miller, 2005). However, for the birth cohorts earlier than 1900 such life tables are not available. Therefore, this study used gender-specific cohort life tables computed from available lifespan data from the Utah Population Database (UPDB) sample (see main text). For computations of the FLoSS for studies from other countries, respective life tables provided by the Human Mortality Database ([www.mortality.org](file:///C:\Users\u0030780\AppData\Local\Microsoft\Windows\INetCache\Content.Outlook\PIRIRTRR\www.mortality.org)) or the Human Life-Table Database ([www.lifetable.de](https://www.lifetable.de)) can be used. These individual scores can be summed up for all deceased family members (i.e., for whom the lifespan information is known) to get the family score. However, such a score would have an undesirable property of increasing with each additional family member, even with those who died at early ages (because the quantities being added would always be positive). Therefore, to avoid this, a constant (1) is subtracted from each individual score which is chosen in such a way that the expected score for a randomly selected person would be 0 (see explanations in (Sebastiani et al., 2009)). Such modified scores are summed for all deceased family members to produce the first component of the FLoSS.

For those family members who are alive, the individual scores look similar but, as their actual lifespans are not known, instead of the achieved age at death the expected age at death is used (i.e., gender- and cohort-specific expected age at death, conditional on survival to the current age of an individual, as computed from respective life tables). Individual scores (with the constant subtracted, similarly to the score for died family members) are summed for all alive family members to produce the second component of the FLoSS.

As the FLoSS is targeted at selecting participants for family longevity studies, families with living siblings should have an additional value because living siblings can provide essential epidemiological, demographic and biospecimen data. Therefore, an additional “bonus” for living siblings is computed in the FLoSS which sums negative logarithms of the probabilities of survival until respective ages (with the constant subtracted, as in the other components of the score) but it does so only for those family members who survived until such ages that respective quantities become positive (so that younger living siblings are not accounted for in the “bonus”). The living siblings’ “bonus” is the third component of the FLoSS and the resulting FLoSS is the sum of the three components described above. See Sebastiani et al. (2009) for additional details and illustration of computations.

# Supplementary Figures and Tables

## Supplementary Figures

| 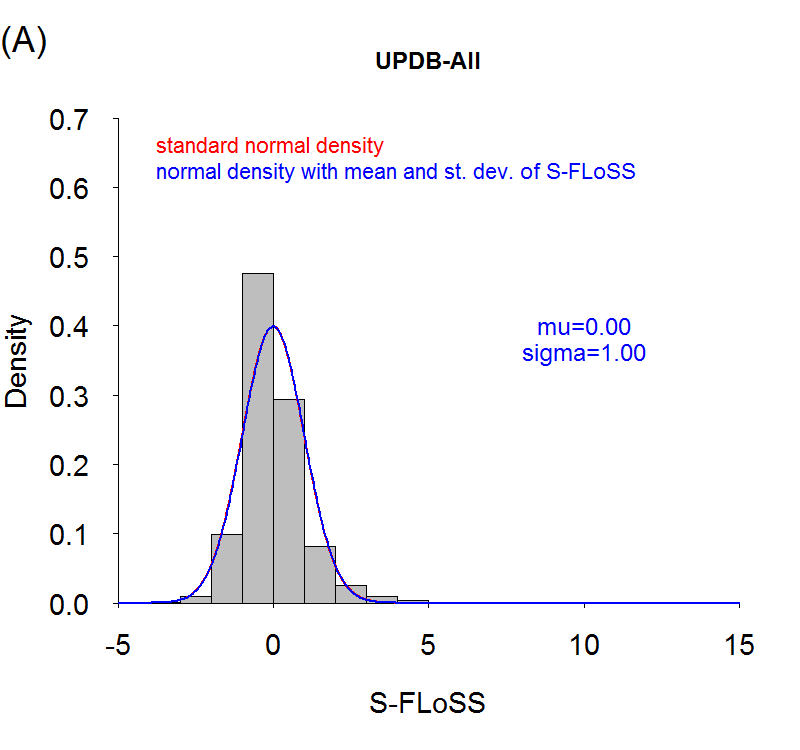 | 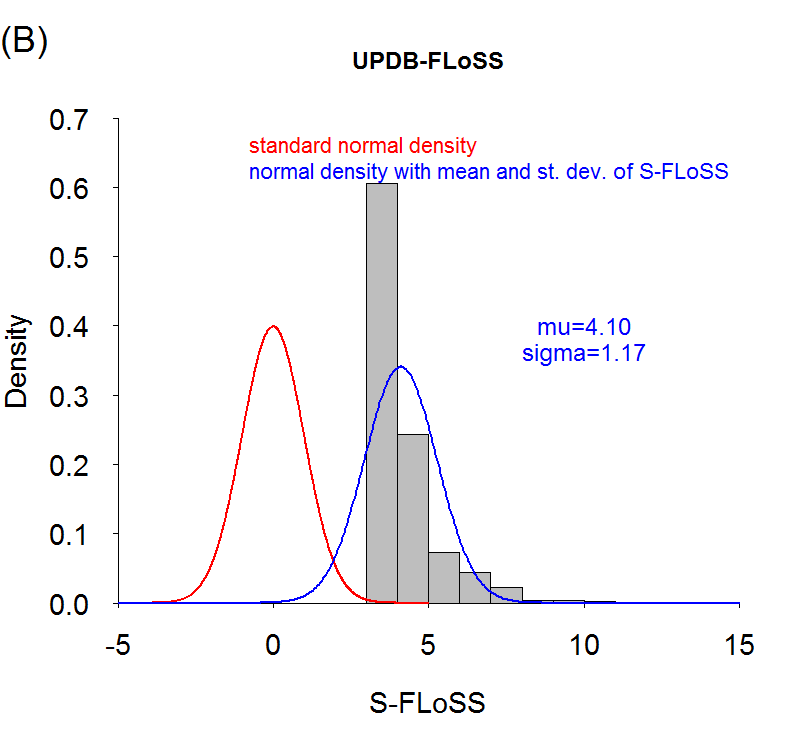 |
| --- | --- |

**Supplementary Figure 1.** Distributions of standardized FLoSS (S-FLoSS) in the UPDB-All **(A)** and the UPDB-FLoSS **(B)** samples. Standardized FLoSS is computed as S-FLoSS=(FLoSS-M)/S, where M=-0.234 and S=2.9 are mean and standard deviation of FLoSS in the UPDB-All sample. The red line shows the standard normal density and the blue line corresponds to the normal density with the mean and standard deviation (denoted as “mu” and “sigma”) of the respective sample. Note that in **(A)** the red and blue lines overlap.


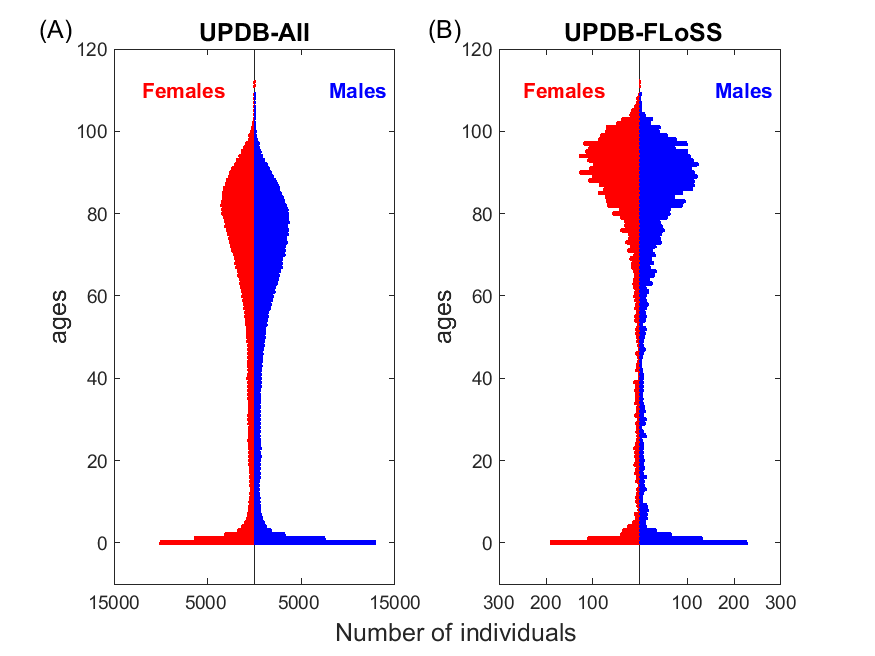


**Supplementary Figure 2.** Distributions of age at death for sibships from the UPDB-All **(A)** and the UPDB-FLoSS **(B)** samples. The figure is based on all individuals with known information on lifespan. Note that although FLoSS is computed using data on individuals surviving until age 40 (see main text), the score is defined at a sibship (not individual) level. That is, the computed score can be assigned to all siblings in a family including those who died before age 40 (or otherwise was not included in computations of FLoSS). Therefore, such individuals appear in panels at ages under 40.

## Supplementary Tables

**Supplementary Table 1.** Description of different samples constructed from the original subset of the Utah Population Database available for this study (“UPDB sample”) and used in the paper

| **Sample** | **Description** | **N_ind_** | **N_sib_** | **Corresponding**  **Figures** |
| --- | --- | --- | --- | --- |
| UPDB-All | Subset from UPDB sample used in FLoSS computations | 234,155 | 57,192 | Figures S1, S2 |
| UPDB-FLoSS | Subset from UPDB-All with FLoSS ≥ 7 | 5,684 | 799 | Figures S1, S2 |
| UPDB-1900 | Subset from UPDB-All with year of birth ≥ 1900 | 29,001 | 16,201 | Figure 1 |
| UPDB-1900-FLoSS | Subset from UPDB-FLoSS with year of birth ≥ 1900 | 647 | 292 | Figure 1 |
| UPDB-1900-AntiFLoSS | Subset from UPDB-All with year of birth ≥ 1900 and FLoSS ≤ -4.5 | 629 | 294 | Figure 1 |
| UPDB-y1980-E | Subset of UPDB-All who were alive and aged 80+ in year 1980 and had FLoSS ≥ 7 | 1,193 | 491 | Figure 2 |
| UPDB-y1980-O | Subset of UPDB-All who were alive and aged 80+ in year 1980 and had FLoSS < 7 | 13,951 | 10,663 | Figure 2 |
| UPDB-1900-EO | Subset from UPDB-All with year of birth ≥ 1900 and with at least one parent having FLoSS ≥ 7 | 993 | 496 | Figure 3 |
| UPDB-1900-OO | Subset from UPDB-All with year of birth ≥ 1900 and with both parents having FLoSS < 7 | 16,541 | 8,922 | Figure 3 |

**Notes: N_ind_** – number of individuals in the sample; **N_sib_** – number of sibships in the sample; “S” in figures denotes “Supplementary”

**References:**

Bell, F.C., and Miller, M.L. (2005). *Life Tables for the United States Social Security Area 1900-2100. Actuarial Study No. 120.* Social Security Administration.

Sebastiani, P., Hadley, E.C., Province, M., Christensen, K., Rossi, W., Perls, T.T., and Ash, A.S. (2009). A Family Longevity Selection Score: Ranking sibships by their longevity, size, and availability for study. *American Journal of Epidemiology* 170**,** 1555-1562.
